# The Role of Probiotics in the Treatment of Vulvovaginal Candidiasis: A Systematic Review and Meta-Analysis

**DOI:** 10.4314/ejhs.v33i5.18

**Published:** 2023-09

**Authors:** Tahere Zahedifard, Talat Khadivzadeh, Marzieh Rakhshkhorshid

**Affiliations:** 1 Ph.D Candidate in Reproductive Health, School of Nursing and Midwifery, Mashhad University of Medical Sciences, Mashhad, Iran. Student Research Committee, Mashhad University of Medical Sciences, Mashhad, Iran; 2 Nursing and Midwifery Care Research Center, Mashhad University of Medical Sciences; Department of Midwifery, School of Nursing and Midwifery, Mashhad University of Medical Sciences, Mashhad, Iran; 3 Ph.D Candidate in Reproductive Health, School of Nursing and Midwifery, Mashhad University of Medical Sciences, Mashhad, Iran; Pregnancy Health Research Center, Zahedan; University of Medical Sciences, Zahedan, Iran

**Keywords:** Probiotic, Lactobacillus, Candidiasis, Vulvovaginal, Meta-Analysis

## Abstract

**Background:**

Vulvovaginal candidiasis is one of the most common vaginal infections worldwide. We conducted this systematic review and meta-analysis to determine the effect of probiotics in the treatment of vulvovaginal candidiasis.

**Methods:**

A comprehensive search of databases including PubMed, Scopus, Cochrane, Scientific Information Database (SID), IranMedex, and Google Scholar search engine was performed. The search was conducted from inception to 1 October 2022, to identify published English or Persian language randomized control trials (RCTs) of women with vulvovaginal candidiasis who received probiotics as medical treatment. The quality of the included studies was assessed using the Oxford Center for Evidence Based Medicine checklist All statistical analyses were performed using Comprehensive Meta-analysis (CMA) version 2.

**Results:**

Six RCTs were included in this review. The results showed that treatment with probiotic was not different from placebo regarding the rate of positive culture (OR: 1.12; 95% CI: 0.390 to 3.26, P=0.825); treatment with probiotic was more effective compared to placebo regarding the rate of recurrence. (OR: 0.14; P= 0.01; 95 % CI: 0.028–0.7).

**Conclusion:**

Probiotics have a beneficial effect in the treatment of women with vulvovaginal candidiasis. Our results provide evidence for an alternative treatment modality for vaginal candidiasis using probiotics.

## Introduction

Vulvovaginal candidiasis (VVC) is one of the most common vaginal infections (1, 2) affecting approximately 75% of women (2, 3). VVC, with a range of symptoms and signs, has a negative impact on women's physical and emotional health as well as sexual and marital relationships (1). The main symptoms of this infection are itching, burning and irritation in the vulvovaginal area associated with the presence of a thick, white vaginal discharge (4). The diagnosis of this type of infection is based on a series of clinical and laboratory symptoms. Diagnostic clinical signs include vulvovaginal pruritus, burning, erythema, edema, and/or curd-like discharge adhering to the vaginal sidewall (5). The laboratory diagnosis of VVC is based on the presence of Candida on wet mount, Gram's stain, or culture of vaginal discharge in a woman along with the aforementioned characteristic clinical findings (6).

Antifungal therapy for VVC is reasonably effective with a number of oral and intravaginal agents, although patients may suffer from its side effect (4). Among the antifungal drugs, azole compounds are considered more convenient than those applied intravaginally (7, 8), so application of oral fluconazole is usually suggested for treatment. Fluconazole maintains therapeutic concentrations in vaginal secretions for at least 72 hours after the ingestion of a single 150-mg tablet (9). Although antifungal agents are effective in the short term, studies have reported the recurrence of infection (recurrent VVC) with a prevalence of more than 50% in the following months (9-10). Usually anti-fungal therapy is not intended to restore the normal vaginal microbiota in patients with recurrent VVC (10-12). In addition, using antifungal agents can lead to bacterial vaginosis (BV), a condition with an overall high prevalence and associated with numerous complications such as preterm birth (13-14) and pelvic inflammatory disease (15). For these reasons, researchers have investigated other treatment approaches to cure or prevent VVC, which may be referred to as probiotics (16).

The word probiotic is derived from the two Greek words pro and bio, meaning life. The Food and Drug Administration defines the word probiotic as live microorganisms that have beneficial effects on host health when administered in adequate amounts (17). Probiotics mainly contain lactic acid bacteria (LAB) strains that stabilize the vaginal microbial balance, so they can be used as a complementary therapy along with conventual treatments to support VCC and prevent recurrence of infection (17-19). According to studies, probiotics may have an indirect effect on healing and preventing recurrence of bacterial vaginosis by secretion. It can also limit the transmission of sexually transmitted diseases (19-20). Another study investigated the effect of probiotic lactobacillus in combination with metronidazole in the treatment of bacterial vaginosis. This study reported an increase in the therapeutic effect of metronidazole (21).

However, conflicting results have been reported regarding the benefit of probiotics in VVC. For example, a study reported a significant reduction in recurrent infection in subjects taking probiotics as prophylaxis compared with a control group (22). Another study conducted in Nigeria used probiotics as adjunctive therapy. This study also reported a reduction in recurrent infection (23). However, in another study, no effect of probiotics in prevention of vulvovaginitis was observed (24). These conflicting results can probably be attributed to the difference in sample size, the presence or absence of a control group, and the administration of probiotics (number and type of probiotics) (25-27). Considering the various contradictory results regarding the probiotic benefits in vaginal candidiasis, the necessity of a meta-analysis study is required to obtain a clear and homogeneous results. Therefore, the aim of this systematic review and meta-analysis is to synthesize the available evidence on the efficacy of probiotics in the treatment of VVC.

## Methods

The present study was conducted based on the preferred reporting items for systematic review and meta-analysis (PRISMA) checklist, but was not registered in the international prospective register of systematic reviews (PROSPERO) database, and a public protocol does not exist. No ethical approval was sought for this systematic review. As this study is a systematic review of previously published studies, the need for ethics approval and patient informed consent was therefore waived.

**Data sources and search strategy**: In this study, a systematic literature review was conducted using the following electronic databases as the most appropriate resources to identify published studies: MEDLINE/PubMed, Scopus, Cochrane, Google Scholar search engine, and Persian databases including Scientific Information Database (SID), IranMedex, and Magiran using equivalent keywords without time restrictions from inception to 1 October 2022. The search terms were (“Vulvovaginal Candidiasis” OR “Vulvovaginal Moniliasis” OR “Vaginitis, Monilial”, OR Vaginitides, OR “Vaginal Yeast Infection” OR Candidiasis AND Probiotic) in the title, abstract, or keywords. All of the aforementioned databases were searched for published, randomized controlled trials (RCTs) in both English and Persian languages. Also, the reference lists of all included studies were hand-searched for any relevant studies missing in the database searches. Six weeks before we submitted the final manuscript to the journal, we performed an updated search on all specified databases. Prior to the search, it was decided that gray literature would not be searched as these studies are not peer-reviewed and lack quality control. In the process of extraction, two investigators independently reviewed both the title of the articles and the abstract to determine their suitability for inclusion.

The selected articles had to meet the following criteria to be included in the review: study participants had to be women in reproductive age (pregnant or non-pregnant), with clinical signs and symptoms of the VVC, whose fungal cultures for candida diagnosis were reported to be positive and received probiotic tablet with or without Azole components for its treatment. Control groups received Azole components alone or placebo. RCTs studies were included, in both English and Persian languages.

**Outcome measure**: The outcome measure in this study was VVC, which was measured through positive culture of candida with the symptoms of VVC or both of them.

**Study selection and data extraction**: EndNote X8 software was used to manage the included studies. Study selection and data extraction were performed by two independent reviewers. Based on the search results, the authors scanned the identified results, abstracts, and relevant records. Full articles of all potentially relevant trials were retrieved. All retrieved studies were screened for multiple publications of the same studies. For each study, we extracted the following data according to a predefined checklist: first author, year and location of the study, study design, participants, intervention, comparison, tools, and outcome. Data were assessed independently by two reviewers, and discrepancies were resolved by discussion with a third researcher. Overall, there was complete agreement between the two reviewers. The summarized characteristics of the included studies are shown in Table 1.

**Table 1:** Characteristics of 6 clinical trials included in study

Author Year and country	Design	Participants	Intervention	Comparison	Tool	Outcome
**Anukam et al (2009) Nigerian**	RCT	Premenopausal women with acute vulvovaginitis(n=59)	One oral dose of fluconazole (150 mg), plus a daily probiotic capsule containing lactobacillus rhamnosus and lactobacillus roteri for 3 months	One oral dose of fluconazole (150 mg), plus one daily placebo capsule	Structured clinical evaluation questionnaire, standard laboratory culture techniques	The results of the study showed that the adjunctive treatment of vulvovaginitis with lactobacillus rhamnosus probiotic and rotari does not affect the recovery rate on the 7th day, but it reduces recurrence. It becomes vulvovaginitis again
**Davar et al. (2016) Iran**	RCT	Patients with candidal vulvovaginitis (number = 59),	The probiotic group (n=28)	Placebo group (n=31)	Fungal culture	The findings demonstrated that taking probiotics with azole antifungal drugs could be highly effective in treating VVC
**Ehrström et al. (2010) Sweden**	RCT	Women (N=95)	Vaginal Capsules containing L gasseri LN40, Lactobacillus fermentum LN99, L. Casei subsp. Rhamnosus LN113 and P. Acidilactici LN23	Placebo	Fungal culture	Vaginal Administration of LN strains after conventional treatment of bacterial vaginosis and/or vulvovaginal candidiasis lead to vaginal colonization, Somewhat fewer recurrences and less malodorous discharge.
**Kovachev et al. (2015) Bulgaria**	RCT	Women between 17 and 50 years (N=436)	Followed the Same treatment schedule; however, ten applications of a Vaginal probiotic containing lactobacillus acidophilus, l. Rhamnosus, streptococcus thermophilus, and l. Delbrueckii Subsp. Bulgaricus were also administered n=209)	150 mg fluconazole and a single vaginal Globule of fenticonazole (600 mg) (n=207)	Microbiological analysis	The local application of probiotics after Administration of combined azoles for treatment of vaginal C. Albicans infections increases therapy efficacy and could Prevent relapse.
**Martinez et al. (2008) Brazil**	RCT	Women diagnosed with VVC	Fluconazole (150 mg) supplemented Every morning for the following 4 weeks with two probiotic Capsules (containing lactobacillus rhamnosus gr-1 and lactobacillus Reuteri rc-14) (n = 29).	Fluconazole (150 mg) supplemented Every morning for the following 4 weeks with two placebo(n = 26)	Microbiological analysis	Probiotic lactobacilli can increase the Effectiveness of an anti-fungal pharmaceutical agent in curing disease
**Pirotta et al. (2004) UK**	RCT, factorial design, Protocol	Women, aged 18–50 years (N=496)	Active oral and Vaginal group (n=124)	Active oral, Vaginal placebo (n = 124), Placebo Oral, active Vaginal (n=124), Placebo Oral and Vaginal (n=124)	Culture of *Candida* species survey	Use of oral or vaginal forms of lactobacillus was not effective in prevention of post-antibiotic vulvovaginitis.

**Figure 1:**
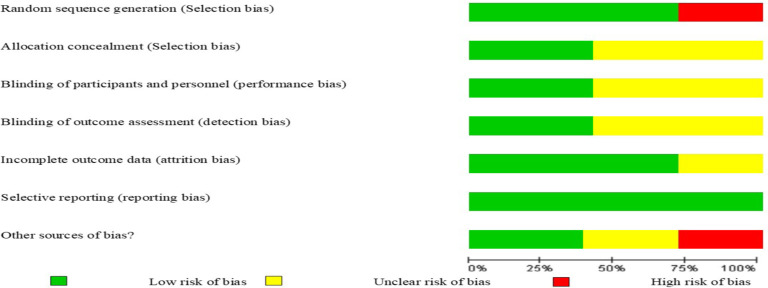
Risk of bias graph: Systematic review. Author's judgments of risk of bias presented as percentages across all included studies

**Quality assessment of the included studies**: The quality of the included studies was assessed using the Oxford Center for Evidence Based Medicine (OCEBM) checklist for RCTs. This tool was designed in two sections to assess internal and external validity. Internal validity in this review was assessed by six general questions including how patients were allocated, similarity and matching of groups, equality of allocated treatment, losses to follow-up and intention to treat analysis, blinding and effect size, which was assessed with three responses of ‘yes’, ‘no’ and ‘unclear’. The assessment of risk of bias is shown in Figure 1. Disagreements between two authors were resolved through consultation with the third author.

**Statistical analysis**: The pooled risk ratio was reported with 95% confidence interval (95% CI). Besides, the randomized model was reported by 95% CI. A p-value < 0.05 was considered statistically significant. The Q statistic and the I^2^ index were used to assess the heterogeneity of the studies. The I^2^ index was used due to its accuracy to compensate for the lack of power (the Q statistic) in small sample sizes or increase the power in large sample sizes. In the I^2^ index, a value below 50%> indicated a low variance in the studies. Moreover, a fixed effect model and the inverse variance method were used. We interpreted the results using effect size and odds ratio (OR). The pooled estimates of OR for VVC diagnosis were calculated. Due to the small number of studies included in the quantitative analysis, funnel plot and Egger test were not performed. All statistical analyses were done using Comprehensive Meta-analysis Version 2 (Biostat, Englewood, NJ, USA).

## Results

**Figure 2:**
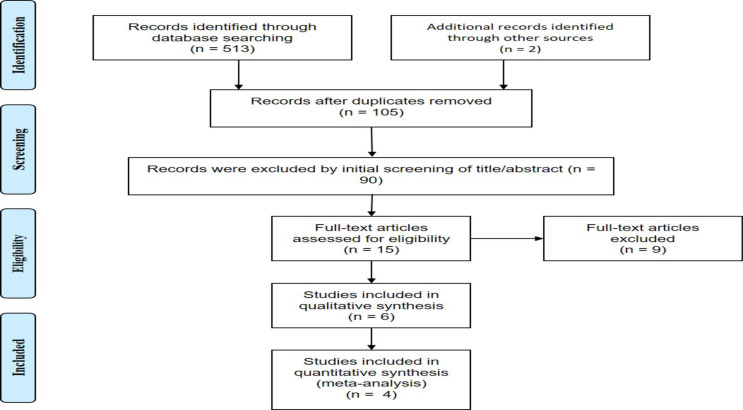
PRISMA Flow Diagram of study selection progress

The database search initially yielded 515 articles, from which six studies were selected for inclusion in the systematic review, and 4 studies were included in the meta-analysis (Figure 2). A total of six RCTs met the inclusion criteria, and four were included in the meta-analysis (Table 1). All the six articles were RCTs designed to evaluate the efficacy of probiotics in VVC patients. A total of 572 patients were assigned to the intervention group and 573 patients to the control group. Of the six trials, one was conducted in Africa (Nigeria), three in Europe (United Kingdom, Bulgaria and Sweden), one in Asia (Iran) and one in Brazil. All studies were published in English. Furthermore, all studies reported the patients receiving probiotics for VVC treatment (Table 1).

**Figure 3:**
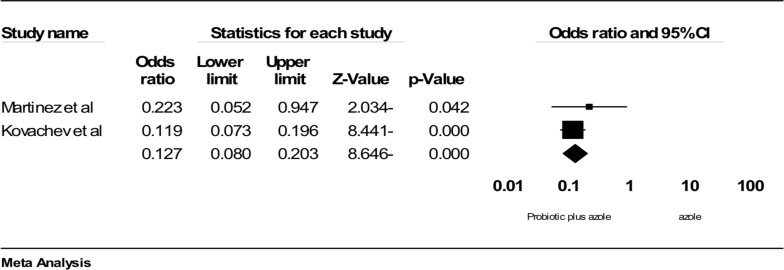
Comparison of the effect of Probiotic in combination azole therapy with alone azole on vaginal culture, ■ point estimate; ♦, combined overall effect of intervention

**Figure 4:**
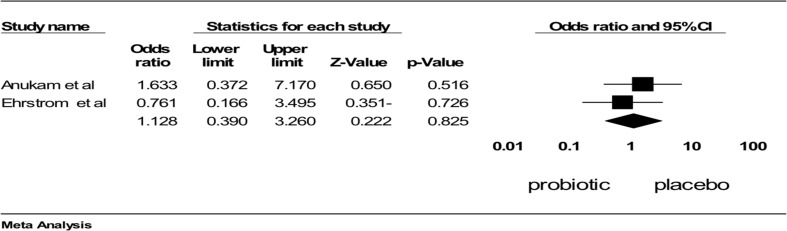
Comparison of the effect of Probiotic compare with placebo on vaginal culture, ■ point estimate; ♦, combined overall effect of intervention

**Comparing the effect of azole therapy plus probiotic versus azole alone on culture and clinical symptoms of VVC**: Two studies had sufficient data to be included in the meta-analysis. Probiotics in combination with azole therapy were found to be effective compared to azole alone in reducing positive culture (OR: 0.106; 95% confidence interval (95%CI): 0.057 to 0.197; p<0.001; heterogeneity: I2=0%, p=0.407, fixed effect model; 2 studies (Figure 3) (34, 35) and clinical symptoms (OR: 1.27 (95% CI: 0.080 to 0.203), p<0.001; heterogeneity: I^2^=0%, p=0.424, fixed effect model; 2 trials; Figure 4) (28-29). In conclusion, it seems that probiotic in combination with azole therapy was found to be effective compared to azole alone and placebo.

**Figure 5:**
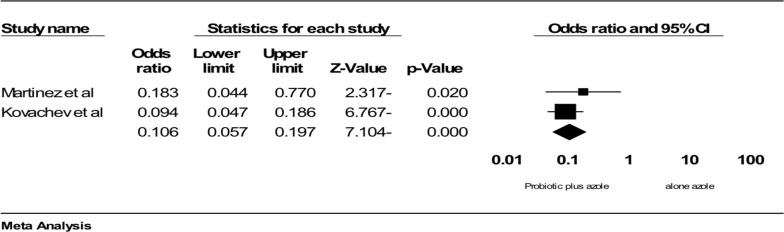
Comparison of the effect of Probiotic compare with placebo on vaginal culture, ■ point estimate; ♦, combined overall effect of intervention

***Comparison of the effect probiotic versus placebo on culture and clinical symptoms of VVC***: Two studies (7,18) compared the effect of probiotic with placebo on vaginal culture. Combining the results of these two trials using meta-analysis showed that treatment with probiotic was not different from placebo regarding the rate of positive culture (OR 1.12 (95% CI: 0.390 to 3.26), p=0.825; heterogeneity: I^2^=0%, p=0.481, fixed effect model; 2 studies; Figure 5). Only one study examined the probiotic effect on clinical compliments. Anukam et al. compared the therapeutic effect of probiotics with that of placebo. They concluded that symptom resolution was higher in patients treated with probiotics than placebo group (47% versus 14%), even though it was non-significant (p=0.279) (23). In summary, it can be concluded that treatment with probiotic was not effective on both positive vaginal culture and clinical symptoms for VVC.

**The effect of probiotic on the recurrence rate of VVC**: Two studies compared the treatment effect of probiotic versus placebo on the recurrence rate of VVC. The first study by Davar et al. (22) compared the recurrence rate of VVC in patients treated with prophylactic probiotic or placebo at 6-month follow-up. The recurrence rate was 0% three months after treatment and 3.6% in the fifth and sixth months in the probiotic group. In the placebo group, the recurrence rate was 6.5% in the first month, 9.6% in the second month, 6.5% in the third month, 0% in the fourth and fifth months, and 12.9% in the sixth month. Comparison of two groups was made using the Fisher's exact test and showed a statistically significant difference regarding the recurrence rate (7.2% for probiotic and 35.5% for placebo. (Odd ratio R 0.14; P = 0.01; 95 %CI (0.028–0.7).

Ehrström, *et al* (30) compared the treatment effect of probiotic with placebo on the recurrence rate of VVC. The recurrence rate was 17% in the placebo group and 7% in the probiotic group two to three days after treatment. The recurrence rate was higher in placebo-treated patients (two to three recurrences in 100% patients) than probiotic-treated patient (one to two recurrence in 53% of patients) at 90 days which is significant at borderline levels. In summary, it seems that treatment with probiotic was more effective compared to placebo in terms of recurrence rate.

## Discussion

In general, the results of the present meta-analysis and review showed that probiotic in combination with azole therapy is more effective compared to azole alone and placebo. However, treatment with probiotic was not effective on both positive vaginal culture and clinical symptoms for VVC. However, it seems that there is lack of control group and failure to report the frequency and percentage, many types of probiotics, great differences among various probiotics, which have great differences in the treatment of VVC, method and time of administration, which have a great impact on the therapeutic effect. Therefore, it is necessary to clarify the issue as much as possible to achieve a definite and practical result. The vaginal area contains a complex and dynamic microbial complex, and lactobacilli are the dominant bacteria in this area. Lactobacilli act as a defense barrier in the urogenital tract and maintain the microbial composition of the vagina in several ways, including competing with other microorganisms (which may have pathogenic potential) for binding to epithelial cells and preventing the growth of pathogens (30-31). In VVC, there is an overgrowth of yeast, most commonly Candida albicans, which normally inhabits the vaginal ecology in low numbers; vaginal lactobacilli levels are not altered in women with VVC (29-30).

Probiotics are crucial in the treatment of vaginitis in women. They inhibit the growth of pathogenic microorganisms by producing a variety of antifungal compounds and lactic acid through lactobacilli, and by stimulating the immune system through competitive adhesion to achieve the effect of treating VVC (31-33). Another advantage of using vaginal probiotics is to reduce the number of pathogens and thus help improve the performance of vaginitis drug treatments by giving rise to metabolites (31). According to meta-analysis results, probiotic in combination with azole therapy was found to be effective compared to azole alone and placebo. Similar to the present study, Reid *et al.* (8), in their study on the effect of prescribing oral Lactobacillus in 64 healthy women, found fungal colonization in 37% of the placebo group compared to 13% of the probiotic group, and the fungal colonization was decreased in the probiotic group (27). In a study by Pirota et al. on the effect of vaginal or oral probiotics on the incidence of VVC after antibiotic treatment, 496 women used oral or vaginal probiotics or both for 4 days. The results showed that taking probiotics after taking antibiotics had no effect on the rate of Candida infection after antibiotic treatment (24).

In contrast, the study by Pirota *et al.* on the effect of vaginal or oral probiotics on the incidence of VVC after antibiotic treatment, in which 496 women used oral or vaginal probiotics or both for 4 days, showed that probiotic use after antibiotic use had no effect on the rate of Candida infection after antibiotic treatment (34). The results of this review on the efficacy of probiotics in alleviating the positive vaginal culture and clinical symptoms of VVC showed that treatment with probiotics was not effective either, which is consistent with the results of studies conducted by other investigators (15, 35). In contrast, in the study by Martins et al., oral probiotics were used for four weeks after treatment with fluconazole. The results showed that the use of probiotics was effective on Candida cultivation. The type of lactobacillus consumed and the duration of use may be one of the reasons for the inconsistency (36). The present study showed that probiotic treatment was more effective compared to placebo in terms of recurrence rate. Some other studies also confirmed these results (37-39). In this study, no side effects were observed in the probiotic recipients. There are some limitations of this systematic review and meta-analysis which should be noted. The existing studies have limited sample size; different selection criteria were utilized in individual studies; different strains and doses of probiotics were used in individual studies; patients experienced different severities of the disease, and finally, our study only analyzed pooled data as no individual patient data was available, restraining comprehensive analyses. Thus, despite the abovementioned outcomes, attention to alternative therapy of VVC, the mechanism of action and advantages of every treatment option like probiotics must be completely investigated before its usage.

In conclusion, this systematic review and meta-analysis found that probiotics were significantly superior to the placebo in treating VVC. Although probiotics showed a favorable effect in the treatment of VVC, more evidence is needed to confirm their effectiveness compared with conventional antifungal treatments. In addition, although the most commonly reported adverse events with probiotics were relatively mild, evidence on safety is still insufficient, and more research is needed.
